# Relationship Between the “Life’s Essential 8” Lifestyle Metrics and Serum High-Sensitivity C-Reactive Protein Levels in South Korea

**DOI:** 10.3390/life16091405

**Published:** 2026-08-25

**Authors:** Seong-Uk Baek, Jin-Ha Yoon

**Affiliations:** 1 Graduate School, Yonsei University College of Medicine, Seoul 03722, Republic of Korea; 2 Institute for Innovation in Digital Healthcare, Yonsei University Health System, Seoul 03722, Republic of Korea; 3 The Institute for Occupational Health, Yonsei University College of Medicine, Seoul 03722, Republic of Korea; 4 Department of Preventive Medicine, Yonsei University College of Medicine, Seoul 03722, Republic of Korea

**Keywords:** cardiovascular health, inflammation, Life’s Essential 8, lifestyle, good health and wellbeing

## Abstract

Background: Mitigation of systemic inflammation may support the achievement of healthy lives and well-being. The American Heart Association recently proposed “Life’s Essential 8” (LE8) lifestyle metric for evaluating cardiovascular health (CVH) status; however, most existing studies have been limited to populations in the United States. This study examined the association between the LE8 score and high-sensitivity C-reactive protein (hs-CRP) levels in Korean adults. Methods: A nationwide sample of 18,500 adults was analyzed. The LE8 score was calculated based on four health behaviors (diet, physical activity, smoking, and sleep health) and four biometric health factors (body mass index, blood lipids, blood glucose, and blood pressure). The LE8 scores were categorized into three groups: 80–100 (ideal CVH), 50–79 (intermediate CVH), and 0–49 (poor CVH). The hs-CRP levels were classified as <3 mg/L or ≥3 mg/L. Logistic regression models were used for the analysis. Results: Among the participants, 21.9%, 67.3%, and 10.9% had ideal, intermediate, and poor CVH, respectively. In the logistic regression models, the adjusted OR (95% CI) for the association between LE8 categories and hs-CRP ≥ 3 mg/L was 1.94 (1.56–2.40) for the intermediate group and 3.21 (2.41–4.28) for the poor group, respectively, compared with the ideal group. Conclusions: Poor CVH status assessed by the LE8 lifestyle health metric was associated with high hs-CRP levels. The study findings suggest that future longitudinal studies are required to establish the temporal relationship between the LE8 and hs-CRP.

## 1. Introduction

The prevention of cardiovascular diseases (CVDs) is a primary public health concern. With the trend of population aging, the number of hospitalizations and mortalities due to CVDs has gradually increased over the past decade [1]. Promoting cardiovascular health (CVH) involves adopting health-promoting lifestyle behaviors and effectively managing chronic conditions such as hypertension, diabetes, and dyslipidemia. Recognizing the importance of promoting CVH, the American Heart Association (AHA) conceptualized the “Life’s Essential 8 (LE8)” framework to facilitate the quantification of individuals’ CVH status and to support risk stratification for targeted interventions [2]. The LE8 framework assesses an individual’s CVH status on a scale of 0–100 based on four health behavior indicators (diet, physical activity, nicotine exposure, and sleep health) and four health factor indicators (blood pressure [BP], body mass index [BMI], blood lipids, and blood glucose). Evidence indicates that a lower LE8 score can predict a higher risk for CVD onset and mortality [3,4,5,6].

Serum high-sensitivity C-reactive protein (hs-CRP) is a well-recognized biomarker for chronic inflammation in both clinical settings and epidemiological investigations. Interleukin-6 (IL-6), among other elevated proinflammatory cytokines during the inflammatory response, stimulates hepatic synthesis and secretion of CRP [7]. Earlier studies demonstrated a positive correlation between hs-CRP levels and the likelihood of CVD onset and mortality [8,9], establishing hs-CRP as a reliable biomarker for CVD risk stratification. For example, the AHA proposed the following risk categories for CVD: hs-CRP levels of 1 mg/L indicated low risk, 1–3 mg/L indicated moderate risk, and levels exceeding 3 mg/L indicated a high risk [10]. Therefore, assessing hs-CRP levels can provide insight into an individual’s chronic inflammatory status and aid in CVD risk stratification.

Examining the association of LE8 with hs-CRP levels can contribute to understanding the role of systemic inflammation in the underlying mechanism [11]. However, few studies have explored this association. Previous studies reported that lower LE8 scores, reflecting a poorer CVH status, are associated with higher serum hs-CRP levels [12,13]. However, research gaps remain in the literature as studies examining the association between LE8 and hs-CRP have primarily focused on Western populations, such as those in the United States [12] or Sweden [13]. To the best of our knowledge, this association has not been reported in Korean or, more broadly, in Asian populations. Compared with Western populations, Asian populations differ substantially in ethnic, genetic, and regional characteristics. For example, Asian populations, including those in South Korea, generally have lower hs-CRP levels than populations in the US or UK [14]. However, Asian populations may have a higher prevalence of certain metabolic risk profiles than Western populations, particularly those in the US [15]. Lifestyle patterns also differ substantially between Asian and Western populations owing to cultural and regional differences [16]. These differences may contribute to variations in LE8 scores and inflammatory biomarker levels, providing a rationale for investigating these associations in South Korea and other Asian populations. As emphasized by the AHA, considering regional and ethnic differences in CVH assessment is essential [2], and research on understudied populations can provide valuable insights into the relevance, applicability, and generalizability of the LE8 framework. Therefore, the present study explored the relationship between LE8 and hs-CRP levels in a nationally representative sample of Korean adults.

## 2. Methods

### 2.1. Study Population

We included a nationwide sample of Korean adults from the 2015–2018 Korea National Health and Nutrition Examination Survey (KNHANES) [17]. The sampling procedure involved multistage systematic sampling to include participants from administrative regions across the entire country. The response rate ranged from 73.1% to 74.1% during the study period. Survey weights were applied in the analyses to ensure representativeness of the sample and mitigate non-response bias. Specifically, sampling weights, strata, and primary sampling units were incorporated in the analysis using Taylor series linearization for variance estimation. No singleton strata were identified during the analysis. For the pooled 2015–2018 dataset, the survey weights were divided by four to account for the combination of four survey years. Written informed consent was obtained from all participants. Raw data from the 2015 to 2018 KNHANES are available at https://knhanes.kdca.go.kr (accessed date: 16 January 2020; access date: 3 March 2024) [17]. The ethical approval was granted by the Institutional Review Board of the Korea Disease Control and Prevention Agency (2018-01-03-P-A).

**Figure 1:**
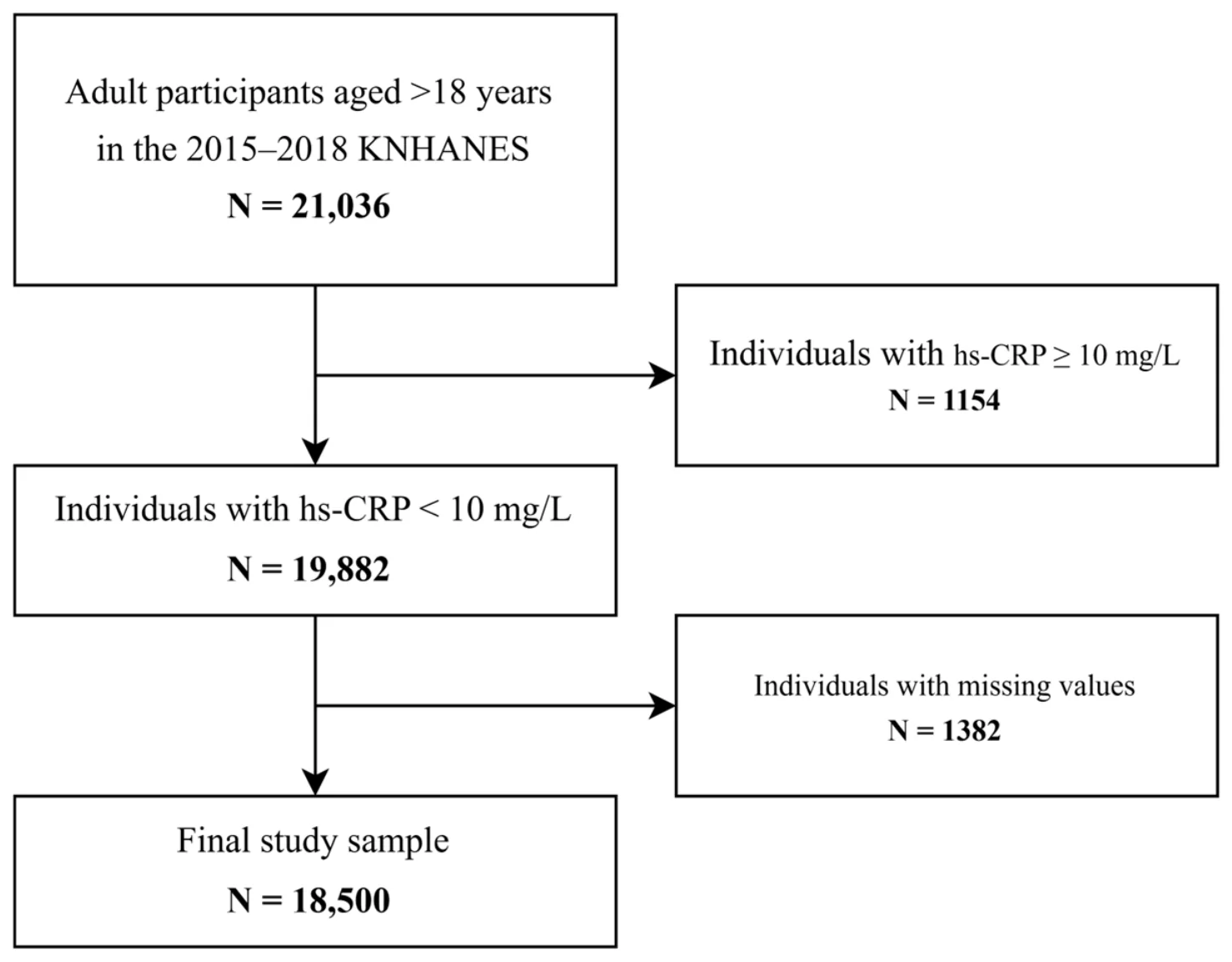
Flowchart of the study sample selection procedure. Adults aged > 18 years who participated in the 2015–2018 KNHANES were included. Individuals with high-sensitivity C-reactive protein (hs-CRP) > 10 mg/L and those with missing values were excluded, resulting in a final study sample of 18,500 participants.

Figure 1 shows the flowchart related to the sample selection. Initially, 21,036 adults aged more than 18 years participated in the 2015–2018 datasets. Subsequently, consistent with the previous study [13], those with hs-CRP levels ≥ 10 mg/L were excluded, as it is indicative of acute inflammatory status or infection. Participants with exactly 10.00 mg/L were excluded. After excluding the participants with missing data, 18,500 adults were included in the final study sample.

### 2.2. LE8 Score

The LE8 score was assessed using eight components comprising four health behaviors and four biometric health factors. The detailed categorization and operationalization of the LE8 scoring system are presented in Table 1. All cutoffs and scoring systems were designed to be consistent with the LE8 algorithm [2].

(1) Health behaviors included diet, physical activity, nicotine exposure, and sleep health. Diet was assessed using the dietary inflammatory index score, which was calculated based on the daily intake of total energy, protein, carbohydrates, fat, iron (Fe), vitamin A, beta-carotene, vitamin B1, vitamin B2, niacin, and vitamin C. Briefly, each dietary factor was transformed into a centered percentile value within the overall distribution, multiplied by its corresponding predetermined inflammatory effect score, and then summed to obtain the total dietary inflammatory index (DII) score. Specifically, each dietary intake was converted into a Z-score. After calculating the Z-scores for each dietary component, they were converted into centered percentile values. Each centered percentile value was then multiplied by the predetermined inflammatory effect score for the corresponding nutrient. The DII score for each individual was calculated as the sum of the component-specific scores across all nutrients [18]. Dietary intake was assessed using 24-hour dietary recall data based on known nutrient composition and recipe information. These data were converted into macronutrient and micronutrient intakes. The detailed calculation method is described in the literature [18]. Consistent with the original scoring system, no adjustment for total energy intake was performed. The percentile categories shown in Table 1 were classified based on the distribution of the overall sample and were aligned with the percentile categories of the AHA dietary components (≤5th, >5–25th, >25–50th, >50–75th, and ≥75th percentiles). In this study, the DII was used because the original DASH score was not assessed in the KNHANES. Second, physical activity was measured using the Global Physical Activity Questionnaire [19]. Weekly moderate-to-vigorous physical activity was calculated as the sum of leisure time and occupational domains. Third, nicotine exposure was evaluated based on current smoking status, duration of smoking history, use of e-cigarettes, and exposure to indoor smoking. Fourth, sleep health was determined by self-reported daily sleep duration.

(2) Biometric health factors include BMI, blood glucose, blood lipids, and BP. Before the health examination, all participants were instructed to fast for at least 8 h overnight. First, BMI (kg/m^2^) was calculated based on weight and height, which were objectively measured. Second, blood glucose levels were determined based on HbA1c (%) and the current use of anti-diabetes or insulin treatment. Third, blood lipid levels were assessed on the basis of non-high-density lipoprotein (HDL) cholesterol levels and the use of lipid-lowering medications. Fourth, BP was determined based on SBP, DBP, and current use of anti-hypertensive medications.

Based on the operationalization described in Table 1, each of the eight LE8 items is scored on a scale ranging from 0 to 100. The overall LE8 score was calculated as the mean of the eight LE8 items. Additionally, mean scores for the health behavior and health factor were calculated. Following the cutoff criteria proposed by the AHA [2], the CVH status was categorized as poor (LE8: 0–49), intermediate (LE8: 50–79), or ideal (LE8: 80–100). Health behavior and factor scores were also categorized as ideal, intermediate, or poor according to the same cutoff values.

### 2.3. hs-CRP

Blood samples were collected by venipuncture and stored under refrigerated conditions at 2–8 °C. hs-CRP levels were measured using an immunoturbidimetric method with a Cobas analyzer (Roche, Mannheim, Germany). Serum hs-CRP levels were recorded to the nearest 0.01 mg/L, and 501 samples with concentrations below the limit of detection (0.15 mg/L) were assigned a value equal to half of the LOD. Following the cutoff widely used in the existing literature [20], the hs-CRP level was categorized as <3 mg/L or ≥3 mg/L.

### 2.4. Confounders

The confounders were selected based on the Directed Acyclic Graph (Figure S1). The following characteristics were adjusted for: sex (male, female), age (years), educational attainment (≤middle school, high school, ≥college education), income level (Q1–Q4), marital status (married, unmarried, other), and employment status (employed, unemployed). The income level was divided into four groups using the quartile values of monthly income for each year.

### 2.5. Statistical Analysis

Logistic regression models were used to assess the association between the LE8 categories and having hs-CRP levels ≥ 3 mg/L. Additionally, the association between each subcategory of the LE8 component (behavior and health factor scores) and hs-CRP levels was analyzed. Subsequently, the nonlinear relationship between continuous LE8 scores and hs-CRP levels was examined using a restricted cubic spline function with four knots, which were placed at the 5th, 35th, 65th, and 95th percentiles. Model-based predicted probabilities were estimated across LE8 scores ranging from 0 to 100 without setting a reference value. Restricted cubic splines were fitted using the rcs() function in the rms package (version 8.1-1). Taylor series linearization was used for variance estimation. For sensitivity analysis, multivariate linear regressions were used to examine the link between the LE8 categories and continuous values of hs-CRP levels. Because the hs-CRP levels exhibited a right-skewed distribution, they were log-transformed to approximate a normal distribution and used as dependent variables. Therefore, the associations between LE8 categories and hs-CRP levels were expressed as percentage (%) changes with corresponding 95% CIs in the sensitivity analyses. Statistical analyses were conducted using R (version 4.6.0). The R package “survey” was employed to apply the complex survey design to the regression models. As a sensitivity analysis, multiple imputation was conducted, in which 20 complete datasets were generated using the multivariate imputation by chained equation method [21].

## 3. Results

The sample characteristics are presented in Table 2. Among the participants, 25.0%, 66.8%, and 8.2% had ideal, intermediate, and poor CVH, respectively (weighted prevalence). Compared to those with ideal CVH (LE8 ≥ 80), those with intermediate or poor CVH tended to be men and older. The mean age for each CVH status group was 41.6, 53.3, and 58.7 years for those with ideal, intermediate, and poor CVH, respectively. Additionally, individuals with poorer CVH were older and exhibited a pronounced socioeconomic gradient, with lower levels of education and income, a higher proportion of those who were not married, and a higher proportion of unemployed individuals. The mean LE8 scores in the overall sample were 69.5, while those were 86.2, 66.5, and 43.2 for those with ideal, intermediate, and poor CVH, respectively. Finally, the prevalence of hs-CRP ≥ 3 mg/L increased progressively as CVH status worsened, from 3.8% in those with ideal CVH to 7.4% and 12.1% in those with intermediate and poor CVH, respectively, indicating a dose–response relationship between CVH status and the prevalence of elevated hs-CRP.

**Figure 2:**
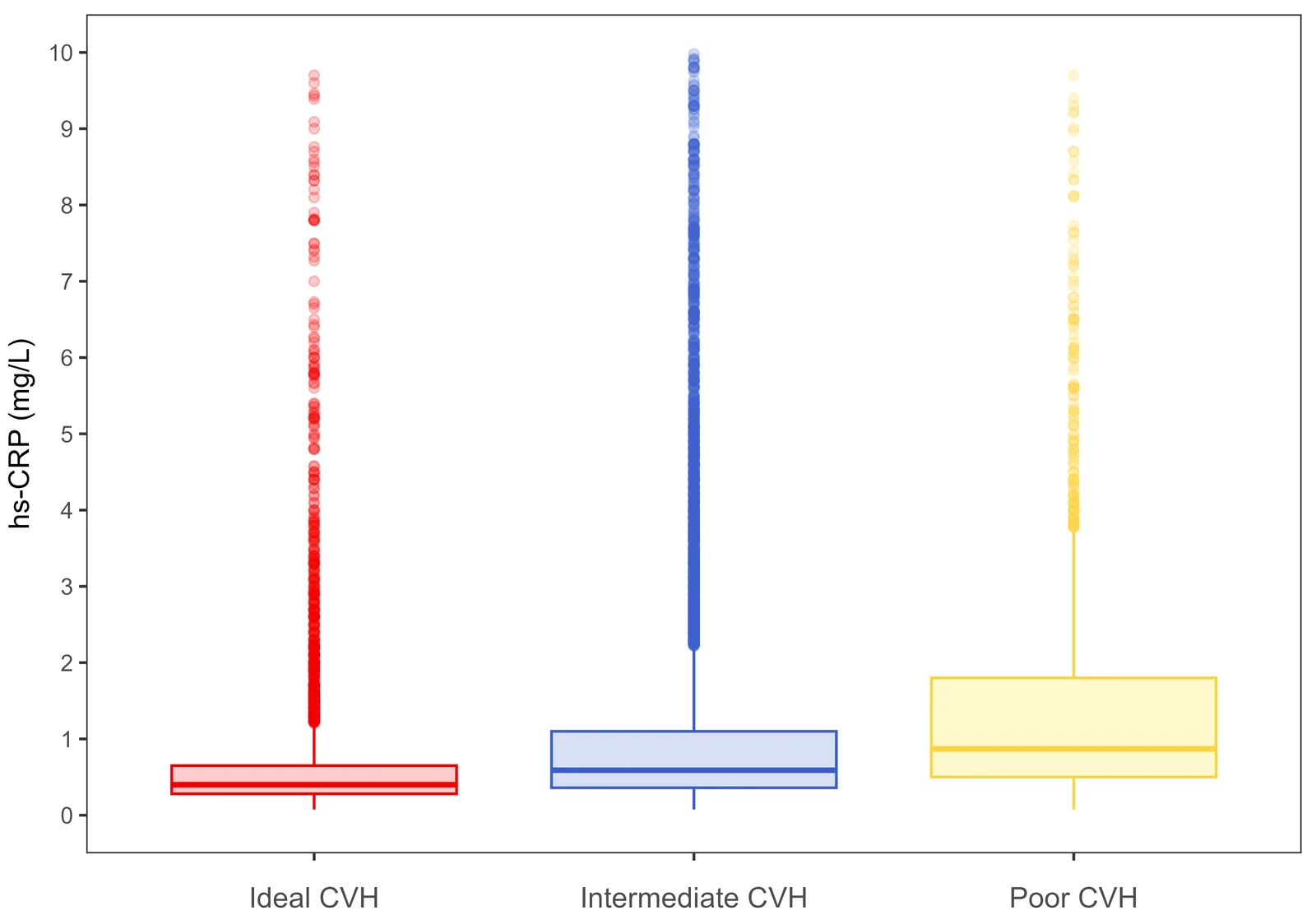
Distribution of hs-CRP levels across LE8 categories.

The distribution of hs-CRP levels across the CVH categories is presented in Figure 2. The median hs-CRP value was 0.50 mg/L in the overall sample, and it was 0.40, 0.60, and 0.87 mg/L for those with ideal, intermediate, and poor CVH, respectively.

In Table 3, the adjusted OR (95% CI) for the association between LE8 categories and hs-CRP ≥ 3 mg/L was 1.94 (1.56–2.40) for the intermediate group and 3.21 (2.41–4.28) for the poor group, respectively, compared with the ideal group. For the LE8 health behavior score, compared to the ideal group, the intermediate or poor group had 1.12-fold (95% CI: 0.95–1.33) and 1.38-fold (95% CI: 1.12–1.70) higher odds of hs-CRP ≥ 3 mg/L, respectively. For the LE8 health factor score, compared to the ideal group, the intermediate or poor group had 1.95-fold (95% CI: 1.61–2.35) and 3.32-fold (95% CI: 2.61–4.22) higher odds of hs-CRP ≥ 3 mg/L, respectively. The adjusted predicted prevalence and absolute difference as well as prevalence ratios drawn from the Poisson regression models were shown in Table S1. Additionally, a sensitivity analysis using a different hs-CRP cutoff (>2 mg/L) also yielded similar results (Table S2). Table S3 presents the results from a model that simultaneously adjusted for the LE8 health behavior and health factor scores, which showed associations similar to those observed in the main models presented in Table 3. Additionally, the results from the model simultaneously including individual LE8 components were shown in Table S4. Finally, the results of the leave-one-component-out analyses are presented in Table S5, consistently demonstrating an association between LE8 and hs-CRP. The sensitivity analysis using multiple imputation also confirmed the association between LE8 and hs-CRP (Table S6).

**Figure 3:**
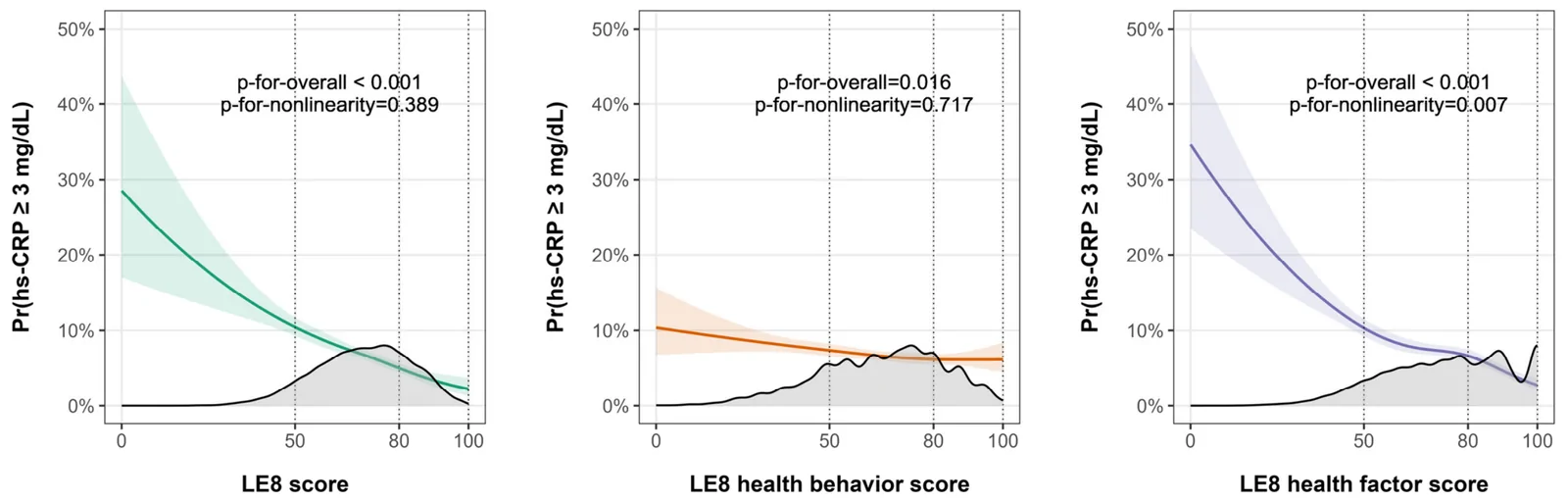
Association between the overall and subdimension scores of LE8 and predicted probability of hs-CRP levels ≥ 3 mg/L using restricted cubic spline function. Models were adjusted for sex, age, education, income, marital status, and employment status.

The nonlinear relationships of overall LE8 score, health behavior, and health factor scores with the predicted probabilities of hs-CRP ≥ 3 mg/L are presented in Figure 3. All three indicators showed negative linear relationships, with the slopes being notably steeper for the overall LE8 and health factors than for health behaviors.

Table 4 presents the relationship between LE8 and its subcategories and hs-CRP levels. In the fully adjusted model, compared to ideal CVH, intermediate CVH was associated with 39.25% (95% CI: 33.66, 45.07) higher hs-CRP levels, while poor CVH was associated with 98.27% (95% CI: 85.79, 111.59) higher hs-CRP levels. For the LE8 health behavior score, compared to the ideal health behavior category, the intermediate health behavior category was associated with 7.88% (95% CI: 3.93, 11.99) higher hs-CRP levels, while the poor health behavior category was associated with 12.29% (95% CI: 6.93, 17.91) higher hs-CRP levels. Finally, for the LE8 health factor score, compared to the ideal health factor category, the intermediate health factor category was associated with 51.45% (95% CI: 46.14, 56.95) higher hs-CRP levels, while the poor health behavior category was associated with 124.82% (95% CI: 111.97, 138.45) higher hs-CRP levels.

## 4. Discussion

This study revealed that the LE8 was associated with hs-CRP levels in the general population in South Korea. Individuals with intermediate or poor CVH were associated with an increased likelihood of having high hs-CRP levels compared to individuals with ideal CVH. Furthermore, biometric health factors exhibited a stronger connection with hs-CRP levels than health behaviors.

Our findings are consistent with those of prior studies linking LE8 to inflammatory markers, including hs-CRP. For instance, studies from Sweden and the US have demonstrated an inverse linear relationship between the LE8 score and hs-CRP levels [12,13]. Hebib et al. (2024) reported that while both health behaviors and factors were associated with hs-CRP levels, the negative association was stronger for health factors than for health behaviors [13], which is consistent with the findings of our study. Additionally, the LE8 score was negatively correlated with the systemic immune inflammation index and white blood cell counts among adults in the United States [22,23]. This study contributes to the existing literature by extending the evidence on the association between LE8 and hs-CRP to a nationally representative Korean population.

Complex mechanisms may underpin the connection between LE8 score and hs-CRP levels. Prior studies have reported that undesirable health behaviors such as poor dietary habits, insufficient physical activity, cigarette smoking, and short sleep duration are linked to elevated hs-CRP levels [24,25,26,27]. Nicotine and toxic substances in tobacco products are known to induce inflammatory responses, whereas antioxidant intake, physical activity, and good sleep quality have been reported to reduce oxidative stress and suppress systemic inflammation [28]. Similarly, poor health factors, including obesity, diabetes mellitus, dyslipidemia, and hypertension, are associated with systemic inflammation and elevated hs-CRP levels [29,30,31]. While previous studies have examined how individual health behaviors and factors contribute to inflammation, the LE8 framework is novel in that it reflects the fact that these CVD risk factors often coexist and collectively influence systemic inflammation rather than acting independently [2].

The present study has some implications for public health policies and clinical practice. Among the various inflammatory markers, hs-CRP is widely recognized as one of the most useful indicators for identifying individuals with chronic low-grade inflammation and predicting CVD onset. A recent scientific statement by the American College of Cardiology emphasized the importance of early lifestyle interventions, including improvements in diet, physical activity, smoking cessation, weight management, and treatment of metabolic risk factors, for individuals with elevated inflammatory risk (hs-CRP ≥ 3 mg/L) [29]. These findings suggest that lifestyle interventions based on the LE8 framework may help mitigate inflammatory conditions and ultimately contribute to both primary and secondary prevention of CVD. From a public health perspective, the LE8 framework provides a valuable basis for monitoring trends in population-level CVH status and developing effective policies that promote healthy lifestyles and reduce CVD-related burdens. Notably, a recent study using a nationally representative Korean sample reported a gradual decline in LE8 scores [32]. Such findings indicate a suboptimal CVH status in the Korean population, which poses a significant public health concern and calls for active policy implementation to improve CVH [33,34].

This study had some limitations. First, a causal link between LE8 and hs-CRP levels could not be established because of the cross-sectional design. The following longitudinal and intervention studies are warranted to examine whether changes in LE8 scores influence hs-CRP levels over time. Second, although hs-CRP is a well-known inflammatory marker used in both epidemiological and clinical research, future studies could benefit from incorporating a broader range of inflammatory cytokines, such as interleukin-6, to provide a more complete evaluation of inflammatory status. Third, as the measurement of health behaviors relies largely on self-reported data, potential recall or social desirability biases may have affected the results. Therefore, the use of objective measurement tools in future follow-up studies should be considered to enhance the accuracy of behavioral assessments. Fourth, DII may conceptually overlap with hs-CRP as a measure of dietary inflammatory potential. However, the association between LE8 and hs-CRP remained robust in the sensitivity analysis after excluding DII from the covariates. Fifth, information on inflammatory disease medications, infections, and other relevant clinical factors was unavailable in the dataset; therefore, sensitivity analyses accounting for these factors could not be performed.

## 5. Conclusions

This study found that the AHA’s LE8 score was associated with serum hs-CRP levels in a nationally representative sample of Korean adults. Future longitudinal studies are needed to confirm the causal relationship between the LE8 score and hs-CRP.

## Figures and Tables

**Table 1:** Operationalization of the Life’s Essential 8 score.

Items	Measurement	Scoring System (Categorization)
1. Diet	Dietary Inflammatory Index (DII) (range: −2.63, 1.45)	100 = <5th %; DII: < −1.47 80 = 5–25th %; DII: −1.47 to −0.32 50 = 25–49th %; DII: −0.32 to 0.32 25 = 50–74th %; DII: 0.32–0.78 0 = ≥75th %; DII: ≥ 0.78
2. Physical activity	min/week of MVPA	100 = ≥150 90 = 120–149 80 = 90–119 60 = 60–89 40 = 30–59 20 = 1–29 0 = 0
3. Nicotine exposure ^a^	Current smoking, past smoking, use of e-cigarettes, exposure to indoor smoking	100 = never smoked 75 = past smoker, quit ≥5 years 50 = past smoker, quit 1–<5 years 25 = past smoker, quit <1 years 25 = current use of e-cigarettes 0 = current smoking
4. Sleep health	Self-reported questionnaire: average hours of sleep per night	100: 7–<9 h 90: 9–<10 h 70: 6–<7 h 40: 5–<6 h 40: ≥10 h 20: 4–<5 h 0: <4 h
5. BMI	Objective measurement of body weight and height (kg/m^2^)	100: <23 75: 23.0–24.9 50: 25.0–29.9 25: 30.0–34.9 0: ≥35
6. Blood lipids ^b^	Total cholesterol, HDL cholesterol, and current use of lipid-lowering agents (mg/L)	Non-HDL cholesterol 100: <130 60: 130–159 40: 160–189 20: 190–219 0: ≥220
7. Blood glucose	HbA1c, current use of oral anti-diabetic medications or insulin	100: No diabetes history and HbA1c < 5.7% 60: No diabetes history and HbA1c 5.7–6.4% 40: Diabetes and HbA1c < 7.0% 30: Diabetes and HbA1c 7.0–7.9% 20: Diabetes and HbA1c 8.0–8.9% 10: Diabetes and HbA1c 9.0–9.9% 0: Diabetes and HbA1c ≥ 10.0%
8. BP ^b^	Approximately sized BP cuff, current use of an anti-hypertensive drug (mmHg)	Systolic BP and diastolic BP 100: <120/<80 75: 120–129/<80 50: 130–139 or 80–89 25: 140–159 or 90–99 0: ≥160 or ≥100
Total LE8	Range: 0–100	80–100: High CVH 50–79: Intermediate CVH 0–49: Low CVH

^a^ Subtract 20 points if exposed to indoor smoking in households. ^b^ Subtract 20 points if drug-treated level. BMI, body mass index; BP, blood pressure; HDL cholesterol, high-density lipoprotein cholesterol; MVPA, moderate-to-vigorous physical activity; e-cigarette, electronic cigarette; HbA1c, hemoglobin A1c.

**Table 2:** Distribution of cardiovascular health status according to study variables.

	Total	CVH Status (LE8 Overall Score)		
Variable		Ideal (80–100)	Intermediate (50–79)	Poor (0–49)
	N = 18,500	N = 4230	N = 12,615	N = 1655
Sex				
Male	7778 (49.8%)	1008 (32.6%)	5618 (53.9%)	1152 (69.3%)
Female	10,722 (50.2%)	3044 (67.4%)	6835 (46.1%)	843 (30.7%)
Age				
Mean (SD)	46.6 (16.3)	38.4 (14.1)	48.9 (16.2)	53.6 (15.2)
Education				
Middle school or lower	5748 (22.3%)	469 (8.4%)	4258 (25.5%)	1021 (39.0%)
High school	5982 (35.8%)	1434 (37. 9%)	3982 (35.1%)	566 (34.3%)
College or higher	6770 (41.9%)	2149 (53.7%)	4213 (39.4%)	408 (26.7%)
Income				
Lowest	4456 (24.1%)	807 (21.2%)	3019 (24.3%)	630 (30.8%)
Low	4620 (24.6%)	963 (23.7%)	3116 (24.8%)	541 (26.2%)
High	4708 (25.4%)	1075 (25.8%)	3167 (25.2%)	466 (25.5%)
Highest	4716 (25.9%)	1207 (29.3%)	3151 (25.6%)	358 (17.5%)
Marital status				
Married	12,952 (65.9%)	2648 (58.1%)	8989 (68.8%)	1315 (66.0%)
Unmarried	2982 (23.7%)	1159 (37.5%)	1652 (19.7%)	171 (14.3%)
Others	2566 (10.4%)	245 (4.4%)	1812 (11.5%)	509 (19.7%)
Employment				
Employed	11,185 (64.5%)	2447 (61.4%)	7614 (65.7%)	1124 (64.0%)
Unemployed	7315 (35.5%)	1605 (38.6%)	4839 (34.3%)	871 (36.0%)
LE8 score				
Mean (SD)	69.5 (13.6)	86.2 (4.7)	66.5 (8.1)	43.2 (5.6)
LE8 health behavior score				
Mean (SD)	64.6 (18.3)	81.1 (9.6)	61.9 (15.4)	37.0 (14.0)
LE8 health factor score				
Mean (SD)	70.9 (19.1)	91.2 (8.9)	71.1 (15.4)	49.3 (13.0)
hs-CRP ≥ 3 mg/L				
No	17,174 (93.1%)	3907 (96.2%)	11,512 (92.6%)	1755 (87.9%)
Yes	1326 (6.9%)	145 (3.8%)	941 (7.4%)	240 (12.1%)

Values are presented as unweighted N (survey-weighted %). CVH, cardiovascular health; SD, standard deviation; hs-CRP, high-sensitivity C-reactive protein.

**Table 3:** Association between the overall and subdimension scores of Life’s Essential 8 and high-sensitivity C-reactive protein levels usign logistic regression models.

	Model 1	Model 2	Model 3
	OR (95% CI)	OR (95% CI)	OR (95% CI)
LE8 overall score			
CVH category			
Ideal	Reference	Reference	Reference
Intermediate	1.99 (1.63–2.42)	1.94 (1.57–2.40)	1.94 (1.56–2.40)
Poor	3.43 (2.67–4.42)	3.31 (2.49–4.41)	3.21 (2.41–4.28)
Continuous scale			
10-point increase	0.76 (0.72–0.80)	0.76 (0.72–0.80)	0.76 (0.72–0.81)
LE8 health behavior score			
Health behavior category			
Ideal	Reference	Reference	Reference
Intermediate	1.16 (0.98–1.37)	1.14 (0.96–1.35)	1.12 (0.95–1.33)
Poor	1.53 (1.25–1.86)	1.44 (1.17–1.77)	1.38 (1.12–1.70)
Continuous scale	0.92 (0.89–0.96)	0.93 (0.90–0.97)	0.94 (0.91–0.98)
10-point increase			
LE8 health factor score			
Health factor category			
Ideal	Reference	Reference	Reference
Intermediate	1.93 (1.64–2.27)	1.95 (1.61–2.35)	1.95 (1.61–2.35)
Poor	3.31 (2.70–4.07)	3.35 (2.64–4.26)	3.32 (2.61–4.22)
Continuous scale			
10-point increase	0.79 (0.77–0.82)	0.79 (0.75–0.82)	0.79 (0.76–0.82)

LE8, Life’s Essential 8; OR, odds ratio; CI, confidence interval. Model 1: unadjusted model. Model 2: sex- and age-adjusted model. Model 3: fully adjusted model.

**Table 4:** Association between the overall and subdimension scores of Life’s Essential 8 and high-sensitivity C-reactive protein levels using linear regression models.

	Model 1	Model 2	Model 3
	% Difference (95% CI)	% Difference (95% CI)	% Difference (95% CI)
LE8 overall score			
CVH category			
Ideal	Reference	Reference	Reference
Intermediate	49.49 (43.87, 55.33)	39.71 (34.15, 45.50)	39.25 (33.66, 45.07)
Poor	123.01 (109.94, 136.89)	100.87 (88.33, 114.24)	98.27 (85.79, 111.59)
Continuous scale			
10-point increase	−17.12 (−18.04, −16.18)	−15.60 (−16.64, −14.54)	−15.54 (−16.60, −14.47)
LE8 health behavior score			
Health behavior category			
Ideal	Reference	Reference	Reference
Intermediate	10.75 (6.66, 15.01)	8.61 (4.65, 12.72)	7.88 (3.93, 11.99)
Poor	24.00 (18.14, 30.15)	14.48 (9.01, 20.22)	12.29 (6.93, 17.91)
Continuous scale			
10-point increase	−4.18 (−5.03, −3.32)	−2.74 (−3.60, −1.87)	−2.38 (−3.25, −1.50)
LE8 health factor score			
Health factor category			
Ideal	Reference	Reference	Reference
Intermediate	57.82 (52.82, 62.98)	51.90 (46.57, 57.42)	51.45 (46.14, 56.95)
Poor	136.36 (123.88, 149.54)	126.34 (113.50, 139.95)	124.82 (111.97, 138.45)
Continuous scale			
10-point increase	−15.72 (−16.42, −15.02)	−15.43 (−16.24, −14.61)	−15.35 (−16.16, −14.52)

LE8, Life’s Essential 8; CI, confidence interval. Model 1: unadjusted model. Model 2: sex- and age-adjusted model. Model 3: fully adjusted model.

## Data Availability

The raw data can be obtained at https://knhanes.kdca.go.kr release date: 16 January 2020; access date: 3 March 2024). The code that supports the findings of this study is available from the corresponding author upon request.

## Supplementary Materials

The following supporting information can be downloaded at: https://www.mdpi.com/article/10.3390/life16091405/s1, Figure S1: DAG on the relationship between LE8 (Life’s Essential 8) score and hs-CRP (high sensitivity C-reactive protein). Table S1: Adjusted predicted prevalence and absolute differences. Prevalence ratios using the fully adjusted Poisson regression models. Table S2: Sensitivity analysis using a different cutoff (hs-CRP > 2 mg/L). Table S3: Results from the model adjusting for the LE8 health behavior and health factor scores simultaneously. Table S4: Results from the model including individual LE8 components simultaneously. Table S5: Results from the leave-one-component-out analyses. Table S6: Association between LE8 and high-sensitivity C-reactive protein levels using multiple imputation. Analytic code (LE8 scoring, survey design code).
