# Supplementary material for: Relationship Between the “Life’s Essential 8” Lifestyle Metrics and Serum High-Sensitivity C-Reactive Protein Levels in South Korea
Source: Life (Basel). 2026 Aug 25;16(9):1405. doi: 10.3390/life16091405 (PMC13608545; doi:10.3390/life16091405)
Supplement: Supplementary file 1 [file life-16-01405-s001.zip › life-4434491-supplementary.pdf]

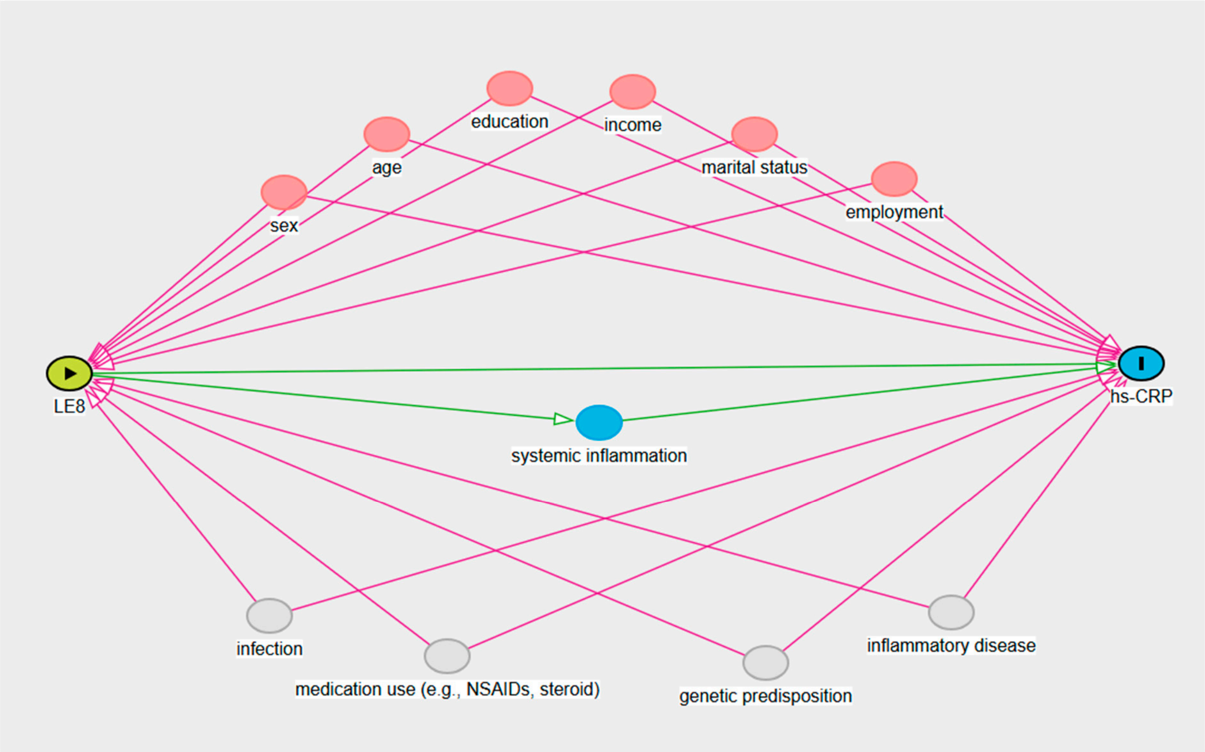

Figure S1. DAG on the relationship between the LE8 (Life's Essential 8) score and hs-CRP (high-sensitivity C-reactive protein).

Table S1. Adjusted predicted prevalence and absolute differences. Prevalence ratios using the fully adjusted Poisson regression models.

	**Adjusted predicted prevalence**	**Absolute difference**	**Prevalence ratio**
**LE8 overall score**			
CVH category			
Ideal	0.04 (0.03–0.05)	Reference	Reference
Intermediate	0.07 (0.07–0.08)	0.03 (0.03–0.04)	1.87 (1.53–2.29)
Poor	0.12 (0.10–0.14)	0.08 (0.06–0.10)	2.95 (2.26–3.84)
**LE8 health behavior score**			
Health behavior category			
Ideal	0.06 (0.05–0.07)	Reference	Reference
Intermediate	0.07 (0.06–0.07)	0.01 (-0.00–0.02)	1.11 (0.95–1.31)
Poor	0.08 (0.07–0.09)	0.02 (0.01–0.04)	1.35 (1.11–1.64)
**LE8 health factor score**			
Health factor category			
Ideal	0.04 (0.04–0.05)	Reference	Reference
Intermediate	0.08 (0.07–0.09)	0.04 (0.03–0.05)	1.87 (1.57–2.24)
Poor	0.13 (0.11–0.15)	0.09 (0.06–0.11)	3.01 (2.42–3.75)

Table S2. Sensitivity analysis using a different cutoff (hs-CRP >2 mg/L).

	Model 1	Model 2	Model 3
	**OR (95% CI)**	**OR (95% CI)**	**OR (95% CI)**
**LE8 overall score**			
CVH category			
Ideal	Reference	Reference	Reference
Intermediate	2.10 (1.80–2.45)	2.04 (1.73–2.40)	2.04 (1.73–2.41)
Poor	3.98 (3.24–4.88)	3.79 (3.03–4.74)	3.70 (2.95–4.64)
Continuous scale			
10-point increase	0.72 (0.70–0.75)	0.72 (0.69–0.75)	0.72 (0.69–0.75)
**LE8 health behavior score**			
Health behavior category			
Ideal	Reference	Reference	Reference
Intermediate	1.24 (1.08–1.42)	1.21 (1.06–1.39)	1.20 (1.04–1.37)
Poor	1.53 (1.31–1.80)	1.42 (1.20–1.67)	1.36 (1.16–1.61)
Continuous scale			
10-point increase	0.92 (0.89–0.95)	0.93 (0.90–0.96)	0.94 (0.91–0.97)
**LE8 health factor score**			
Health factor category			
Ideal	Reference	Reference	Reference
Intermediate	2.05 (1.81–2.32)	2.09 (1.81–2.41)	2.09 (1.82–2.42)
Poor	4.30 (3.65–5.07)	4.42 (3.67–5.33)	4.39 (3.64–5.30)
Continuous scale			
10-point increase	0.75 (0.73–0.77)	0.74 (0.72–0.77)	0.74 (0.72–0.77)

Table S3. Results from the model adjusting for the LE8 health behavior and health factor scores simultaneously.

	Model 1	Model 2	Model 3
	**OR (95% CI)**	**OR (95% CI)**	**OR (95% CI)**
**LE8 health behavior score**			
Health behavior category			
Ideal	Reference	Reference	Reference
Intermediate	1.10 (0.93–1.30)	1.10 (0.93–1.30)	1.09 (0.92–1.29)
Poor	1.35 (1.11–1.65)	1.37 (1.12–1.68)	1.33 (1.08–1.64)
Continuous scale			
10-point increase	0.95 (0.91–0.98)	0.94 (0.91–0.98)	0.95 (0.91–0.99)
**LE8 health factor score**			
Health factor category			
Ideal	Reference	Reference	Reference
Intermediate	1.89 (1.61–2.22)	1.93 (1.60–2.34)	1.94 (1.61–2.35)
Poor	3.21 (2.62–3.95)	3.30 (2.60–4.19)	3.29 (2.59–4.18)
Continuous scale			
10-point increase	0.80 (0.77–0.83)	0.79 (0.76–0.82)	0.79 (0.76–0.83)

Table S4. Results from the model including individual LE8 components simultaneously.

	Model 1	Model 2	Model 3
	**OR (95% CI)**	**OR (95% CI)**	**OR (95% CI)**
**LE8 components**			
Diet	0.99 (0.96–1.01)	0.99 (0.97–1.01)	0.99 (0.97–1.02)
Physical activity	0.99 (0.97–1.00)	0.99 (0.98–1.01)	0.99 (0.97–1.01)
Nicotine exposure	0.98 (0.96–1.00)	0.97 (0.95–0.99)	0.97 (0.95–0.99)
Sleep	1.00 (0.97–1.02)	1.00 (0.97–1.03)	1.00 (0.97–1.03)
Body mass index	0.84 (0.81–0.86)	0.83 (0.81–0.86)	0.84 (0.81–0.86)
Blood pressure	1.00 (0.97–1.02)	1.00 (0.98–1.02)	1.00 (0.97–1.02)
Blood glucose	0.94 (0.92–0.97)	0.95 (0.92–0.98)	0.95 (0.92–0.98)
Blood lipids	0.99 (0.97–1.01)	0.99 (0.97–1.02)	0.99 (0.96–1.01)

Table S5. Results from the leave-one-component-out analyses.

	Model 1	Model 2	Model 3
	**OR (95% CI)**	**OR (95% CI)**	**OR (95% CI)**
**LE8 components**			
-Diet	0.77 (0.74–0.81)	0.77 (0.73–0.81)	0.76 (0.73–0.81)
-Physical activity	0.75 (0.71–0.78)	0.74 (0.70–0.79)	0.75 (0.70–0.79)
-Nicotine exposure	0.77 (0.74–0.81)	0.77 (0.73–0.81)	0.78 (0.73–0.82)
-Sleep	0.78 (0.74–0.81)	0.77 (0.73–0.81)	0.77 (0.73–0.82)
-Body mass index	0.82 (0.78–0.86)	0.83 (0.79–0.87)	0.83 (0.79–0.88)
-Blood pressure	0.77 (0.73–0.81)	0.77 (0.73–0.82)	0.78 (0.73–0.82)
-Blood glucose	0.79 (0.75–0.82)	0.79 (0.75–0.83)	0.79 (0.75–0.84)
-Blood lipids	0.77 (0.74–0.81)	0.77 (0.73–0.82)	0.78 (0.74–0.82)

Table S6. Association between LE8 and high-sensitivity C-reactive protein levels using multiple imputation.

	Model 1	Model 2	Model 3
	**OR (95% CI)**	**OR (95% CI)**	**OR (95% CI)**
**LE8 overall score**			
CVH category			
Ideal	Reference	Reference	Reference
Intermediate	2.00 (1.64–2.44)	1.95 (1.57–2.41)	1.87 (1.53–2.29)
Poor	3.46 (2.69–4.45)	3.32 (2.50–4.42)	3.22 (2.42–4.30)
**LE8 health behavior score**			
Health behavior category			
Ideal	Reference	Reference	Reference
Intermediate	1.12 (0.94–1.32)	1.12 (0.94–1.32)	1.12 (0.94–1.32)
Poor	1.38 (1.12–1.70)	1.38 (1.12–1.70)	1.38 (1.12–1.70)
**LE8 health factor score**			
Health factor category			
Ideal	Reference	Reference	Reference
Intermediate	1.93 (1.64–2.26)	1.96 (1.63–2.35)	1.96 (1.63–2.36)
Poor	3.40 (2.79–4.16)	3.47 (2.75–4.37)	3.43 (2.72–4.33)

Analytic code (LE8 scoring, survey design code)

LE8 score code

```
Data <- Data %>%
  mutate(

    diet.score=case_when(
      DII < -1.47353072 ~ 100,
      DII >= -1.47353072 & DII < -0.32100441 ~ 80,
      DII >= -0.32100441 & DII < 0.31977472 ~ 50,
      DII >= 0.31977472 & DII < 0.77686265    ~ 20,
      DII >= 0.77686265    ~ 0
    ),

    pa.score=case_when(
      Total==0 ~ 0,
      Total>0 & Total <30 ~ 20,
      Total>=30 & Total <60 ~ 40,
      Total>=60 & Total <90 ~ 60,
      Total>=90 & Total <120 ~ 80,
      Total>=120 & Total <150 ~ 90,
      Total>=150 ~ 100,
    ),

    nicotine.score=case_when(
      BS3_1 %in% c(1,2) ~ 0,
      BS3_1 ==3 & BS6_4_1 %in% c(0) ~ 25,
      BS3_1 %in% c(3,8) & BS12_2 ==1 ~ 25,
      BS3_1==3 & BS6_4_1 %in% c(1:4) ~ 50,
      BS3_1==3 & BS6_4_1 %in% c(5:70) ~ 75,
      BS3_1==8 & BS12_2 %in% c(8) ~ 100,
    ),

    indoor=case_when(
      BS9_2 ==1 ~ 1,
      BS9_2 %in% c(2,3,8) ~ 0
    ),

    nicotine.score2=case_when(
      nicotine.score==0 | indoor==0 ~ nicotine.score,
      nicotine.score %in% c(25,50,75,100) & indoor ==1 ~ nicotine.score-20
    ),

    sleep.score=case_when(
      sleepdu >0 & sleepdu <4 ~0,
      sleepdu >=4 & sleepdu<5 ~ 20,
      sleepdu >=5 & sleepdu<6 ~ 40,
      sleepdu >=6 & sleepdu<7 ~ 70,
      sleepdu >=7 & sleepdu<9 ~ 100,
      sleepdu >=9 & sleepdu<10 ~ 90,
      sleepdu>=10 & sleepdu<16 ~ 40,
    ),

    BMI.score=case_when(
      HE_BMI<23 ~ 100,
      HE_BMI>=23 & HE_BMI<25 ~ 75,
```

```
    HE_BMI>=25 & HE_BMI<30 ~ 50,
    HE_BMI>=30 & HE_BMI<35 ~ 25,
    HE_BMI>=35 ~ 0,
),

nonhdl=HE_chol-HE_HDL_st2,
lipid.score=case_when(
    nonhdl<130 ~ 100,
    nonhdl>=130 & nonhdl<160 ~ 60,
    nonhdl>=160 & nonhdl<190 ~ 40,
    nonhdl>=190 & nonhdl<220 ~ 20,
    nonhdl>=220 ~ 0,
),

lipid.score2=case_when(
    lipid.score %in% c(20,40,60,100) & DI2_pt==1 ~ lipid.score-20,
    lipid.score==0 | DI2_pt %in% c(0,8) ~ lipid.score
),

glucose.score=case_when(
    DE1_pr %in% c(0,8) & HE_HbA1c <5.7 ~ 100,
    DE1_pr %in% c(0,8) & HE_HbA1c >=5.7 & HE_HbA1c <6.5 ~ 60,
    DE1_pr %in% c(1) & HE_HbA1c <6.5 ~ 40,
    HE_HbA1c >=6.5 & HE_HbA1c<7 ~ 40,
    HE_HbA1c >=7 & HE_HbA1c<8 ~ 30,
    HE_HbA1c >=8 & HE_HbA1c<9 ~ 20,
    HE_HbA1c >=9 & HE_HbA1c<10 ~ 10,
    HE_HbA1c >=10 ~ 0
),

bp.score=case_when(
    HE_sbp<120 & HE_dbp<80 ~ 100,
    HE_sbp %in% c(120:129) & HE_dbp<80 ~ 75,
    HE_sbp %in% c(130:139) & HE_dbp<90 ~ 50,
    HE_sbp <140 & HE_dbp %in% c(80:89) ~ 50,
    HE_sbp %in% c(140:159) & HE_dbp<100 ~ 25,
    HE_sbp <160 & HE_dbp %in% c(90:99) ~ 25,
    HE_sbp >=160 | HE_dbp >= 100 ~ 0
),

bp.score2=case_when(
    bp.score %in% c(25,50,75,100) & DI1_pt==1 ~ bp.score-20,
    bp.score ==0 | DI1_pt %in% c(0,8) ~ bp.score,
),

life8score=(diet.score + pa.score + bp.score2 + lipid.score2 + glucose.score + BMI.score +
                        nicotine.score2+sleep.score)/8,

behaviorscore=(diet.score + pa.score +nicotine.score2+sleep.score)/4,

factorscore=(bp.score2 + lipid.score2 + glucose.score + BMI.score )/4,

LE8_cat=case_when(
    life8score<50 ~ "c",
    life8score>=50 & life8score <80 ~ "b",
    life8score >=80 ~ "a",
),

Behavior_cat=case_when(
```

```
                        behaviorscore <50 ~ "c",
                        behaviorscore >=50 & behaviorscore <80 ~ "b",
                        behaviorscore >=80 ~ "a",
                    ),

                    Factor_cat=case_when(
                        factorscore <50 ~ "c",
                        factorscore >=50 & factorscore <80 ~ "b",
                        factorscore >=80 ~ "a",
                    ),
    )
```

```
# survey design code

data_svy<-svydesign(data=data, strata=~kstrata, ids=~psu, weights =~ wt)
```
